# Carotid Artery Calcification: A Digital Panoramic-Based Study

**DOI:** 10.3390/diseases6010015

**Published:** 2018-02-08

**Authors:** Ibrahim Nasseh, Georges Aoun

**Affiliations:** Department of Oral Medicine and Maxillofacial Radiology, Faculty of Dental Medicine, Lebanese University, Beirut, Lebanon

**Keywords:** calcification, carotid artery, Lebanese, panoramic radiography, population

## Abstract

Objective: The aim of this study was to estimate the incidence of carotid artery calcification (CAC) in a sample of Lebanese population using digital panoramic radiographs. Materials and Methods: Panoramic radiographs of 500 patients (281 females and 219 males), aged between 18 and 88 years (mean: 47.9 years), were assessed for CAC. Data collected were analyzed statistically using IBM^®^ SPSS^®^ for Windows version 20.0 (SPSS, Chicago, IL, USA). Results: CAC were found in 34 cases (6.8%), among them, 23 females (8.18%) and 11 males (5.02%). Six of all the calcifications were on the right side, against six on the left side, and 22 on both sides. The mean age of patients affected with CAC was 60.9 years (ranging from 18 to 88 years). Chi-square test showed no statistical significance between gender and CAC, while Spearman correlation analysis showed positive low correlation with age (*r* = 0.179). Conclusion: CAC can be found on routine panoramic radiographs taken in dental clinics; dentists should automatically refer the patients in question for specialized medical evaluation.

## 1. Introduction

The accumulation of atheromas, which are calcified plaques composed essentially of fatty substances, macrophage cells, lipids, calcium, fibrous connective tissue, etc., in the walls of the carotid arteries, may lead to a cerebrovascular accident [1,2,3].

According to many studies, cerebrovascular accidents represent the third cause of death worldwide; moreover, and about 60% of the surviving patients would suffer from mental and/or physical disabilities [4,5,6,7,8,9].

Therefore, knowing that atheromas are generally located in the bifurcation of the common carotid artery, and that the early detection of these calcifications may help decrease cerebrovascular accidents incidence considerably, there has been increased awareness in radiologic investigation as a noninvasive way to trace them [3,10,11,12].

Friedlander and Lande [13], followed by many other researchers, identify carotid artery calcification (CAC) by means of conventional imaging techniques used in dental practice, e.g., panoramic radiography.

**Figure 1:**
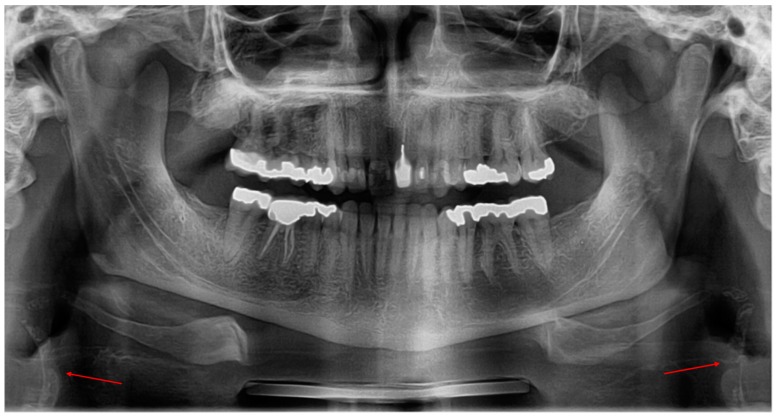
Bilateral carotid artery calcification.

Carotid artery calcification was described, radiographically, as irregular nodular radiopacity located posteroinferiorly to the mandibular angle and the hyoid bone, adjacent to the cervical vertebrae, close to the intervertebral space C3–C4 [10,11,14] (Figure 1). Nevertheless, because CAC looks like other soft tissue calcifications located in the same radiologic region, its diagnosis is sometimes problematic; among these calcifications is the calcified triticeous cartilage.

With the absence of any radiologic exploration for CAC in Lebanon, the aim of this study was to assess this type of calcification in a Lebanese sample via panoramic radiography.

## 2. Material and Methods

This retrospective study assessed archived digital panoramic radiographs of Lebanese adult patients, taken initially for dental diagnosis reasons in a specialized maxillofacial radiology center in Beirut, Lebanon.

According to the center policy, all patients were informed that their radiographs may be anonymously used for research purposes, and their approval was obtained.

All panoramic radiographs taken were done with the Pax Zenith digital panoramic unit (Vatech, Korea). The settings of the X-ray unit were selected according to the patient profile (60–90 kV, 6–10 mA). Exposure time was 10–20 s.

The exclusion criteria included the lack of patients’ information (age and gender), and low-quality images and radiographs not showing C3–C4 vertebrae.

Five hundred (500) panoramic radiographs of 219 males and 281 females, aged between 18 and 88 years, were selected and examined by an oral and maxillofacial radiologist with more than 20 years of experience, on the same monitor. The research procedure extended over five sessions, spaced by a fifteen day period.

Furthermore, to decrease errors, the primary exploration was repeated two weeks later, without having in hand, the initial results.

**Figure 2:**
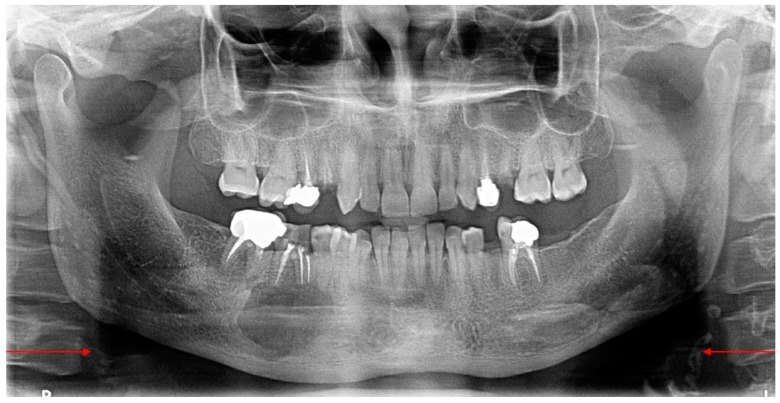
Panoramic image showing the presence of multiple small radiopaque entities inferior to the angle of the mandible at the level of C3–C4.

Carotid artery calcifications were identified as irregular uni—or bilateral radiopaque nodular mass/masses, curvy—or roughly verticolinear, located inferiorly to the mandibular angle, adjacent to the intervertebral space C3–C4 (Figure 2).

Data entry and analyses were performed using IBM^®^ SPSS^®^ for Windows version 20.0 (SPSS, Chicago, IL, USA). Descriptive statistics of age, gender, and CAC were calculated. Chi-square test and Spearman correlation analysis were used to test statistical significance. Statistical significance was set at 0.05.

## 3. Results

**Figure 3:**
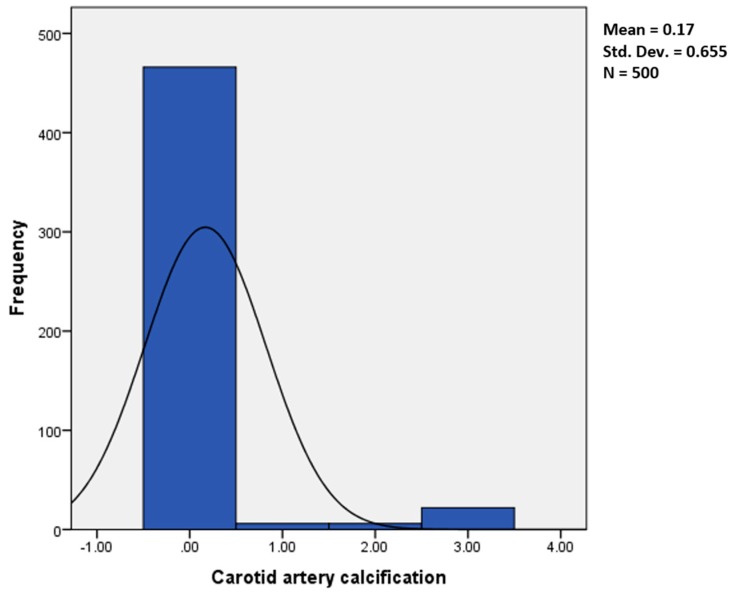
Frequency of CAC in our sample (0 denotes absence, 1 denotes right side, 2 denotes left side and 3 denotes bilateral formation).

The study population consisted of 500 patients (219 males and 281 females) aged 18–88 (mean: 47.9 years). In our sample, CAC was seen in 34 cases (6.8%) (23/281 females and 11/219 males); among these, 6 (1.2%) (4 females and 2 males) were on the right side, 6 (1.2%) (5 females and 1 male) on the left, and 22 (4.4%) (14 females and 8 males) were bilateral (Figure 3).

When assessing the association gender–CAC, Chi-square test showed no statistically significant relation between gender and CAC (*p* = 0.1).

**Figure 4:**
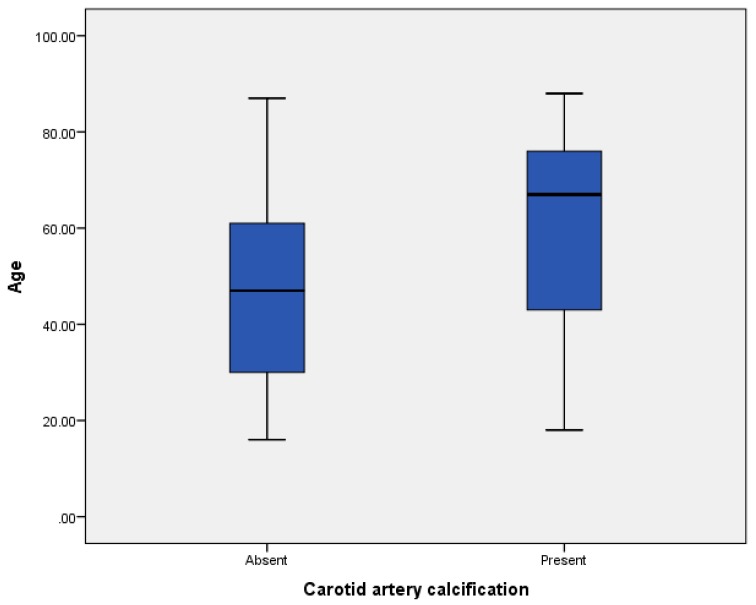
CAC–age in our sample.

In our sample, the mean age of patients affected with CAC was 60.9 years (ranging from 18 to 88 years) (Figure 4). Spearman correlation analysis showed a positive low correlation between age and CAC (*r* = 0.179).

## 4. Discussion

Head and neck calcifications are frequently found in patients seeking dental care. Usually asymptomatic, they may be fortuitously detected on panoramic dental radiographs [1,3,4,5,6,7,8,9]; among these calcifications, CAC is one of the major causes of cerebrovascular accidents.

The incidence of CAC detected on panoramic radiographs was investigated by many authors in different populations [15,16,17,18,19,20,21,22].

In our study, conducted on a sample of the Lebanese population, the prevalence of CAC was 6.80%; it was age-dependent (mean = 60.9 years). These findings support the ones of Lee et al., who studied CACs in a South Korean population using the same method (6.2% of the patients older than 50 years) [17]. However, the authors admitted that their results were higher than the ones reported by previous studies conducted on Asian populations; Pornprasertsuk-Damrongsri and Thanakun [18], and Kumagai et al. [19] found, respectively, an incidence of 2.5% (Thai) and 4% (Japanese).

Besides these examples, lower findings were also reported by Bayram et al. [1] (2.1% in Turkish), Alzoman et al. [15] (5% in Saudi), and Abreu et al. [22] (2.9% in Brazilian).

On the other hand, other studies noticed higher occurrences of CAC than ours (Brand et al. [21], 9.4% in Dutch; Moshfeghi et al. [3], 11% in Iranian; Uthman and Al-Saffa [20], 38.8% in Iraqi).

This large variation between the different studies might be related to dietary habits, lifestyles, and the sample sizes and types (age, genders, etc.).

It is important to note that all these studies have in common the close relationships CAC–patient’s age and CAC–patient medically at risk.

According to Scarfe and Farman [23], the incidence of CAC detected on panoramic images varies between 0.1% and 3.2% in patients aged 50 years and older, and rises in populations at high atherosclerotic risk (smokers, obese, inactive, suffering from hypercholesterolemia, etc.).

The high morbidity and mortality rates caused by CAC necessitate, if suspected, urgent patient referral to a specialist physician, to confirm the potential arterial damage intimately linked to cerebrovascular accidents [1,5,11].

Interestingly, Yeluri et al., in their study, interconnect the presence of pulp stones and CAC; consequently, they declared that an advanced arterial assessment must be done when multiple pulp stones are noticed [24].

Finally, our study aiming to estimate the occurrence of CAC in a sample of Lebanese population is not without limitations. Although most radiologic assessments were made with high reliability, the limited number of patient radiographs reviewed makes essential the investigation on a larger group, which can lead to more accurate results.

## 5. Conclusions

Panoramic radiographs may have some diagnostic importance for detecting CAC closely connected to atherosclerosis, thus preventing major life-threatening events such as cerebrovascular accidents.
